# Idiopathic intracranial hypertension in the British population with obesity

**DOI:** 10.1007/s00701-018-3772-9

**Published:** 2018-12-18

**Authors:** Maddalena Ardissino, Osama Moussa, Alice Tang, Elisabetta Muttoni, Paul Ziprin, Sanjay Purkayastha

**Affiliations:** 1 Department of Medicine, Imperial College London, London, UK; 2 Academic Unit, Department of Surgery and Cancer, St Mary’s Hospital, Imperial College Healthcare NHS Trust, London, UK; 3 St. Helens and Knowsley Teaching Hospitals, Whiston Hospital NHS Trust, Liverpool, UK

**Keywords:** Body mass index, BMI, Vision, Neuropathology, Obesity, Intracranial hypertension

## Abstract

**Purpose:**

Idiopathic intracranial hypertension (IIH) is a syndrome that is characterized by persistently high intracranial pressure and associated with high rates of morbidity and visual loss. Its exact etiology and clinical picture is poorly understood, but it is known to be associated with obesity. The aim of this study was to investigate the prevalence and clinical manifestations of IIH using a large nationwide database of British subjects.

**Materials and methods:**

The anonymized healthcare records of patients with a BMI of ≥ 30 kg/m^2^ were extracted from the Clinical Practice Research Datalink (CPRD), and analyzed.

**Results:**

The patients with IIH were older and more likely to have peripheral vascular disease, ischemic heart disease, and anemia; to have had a previous myocardial infarction; and have used non-steroidal anti-inflammatory drugs (NSAIDs) and steroids. Multivariate analysis with adjustment for confounders showed that anemia (*p* = 0.033) and the use of NSAIDs (*p* = 0.011) were the only factors independently associated with IIH. Increases in BMI beyond the threshold of obesity did not independently increase risk of IIH.

**Conclusions:**

IIH is a multifactorial disease; the risk of which is increased in patients with a background of anemia, and those who use NSAIDs. Across BMI categories beyond the threshold for obesity (BMI ≥ 30 kg/m^2^), there is no continuation of the previously described “dose-response” relationship between BMI and IIH.

**Ethical approval:**

Scientific approval for the study was granted from the Regulatory Agency’s Independent Scientific Advisory Committee and ethical approval by the Health Research Authority IRAS Project ID: 203143. ISAC approval registration number 16_140R2.

## Introduction

### Background

Idiopathic intracranial hypertension (IIH) is a complex condition that is defined as persistently high intracranial pressure in the absence of any detectable cerebrospinal fluid; brain parenchymal, meningeal, or cerebral vein pathology; and diagnosed using the modified Dandy criteria [4]. Its etiology is still obscure, but it classically presents with persistent headache, nausea, photophobia, tinnitus, and visual disturbances (which may begin as mild visual dysfunction but can rapidly progress to complete visual loss). It can therefore cause significant morbidity if left untreated [5]. It is relatively rare, affecting 0.9/100,000 people per year, but has been reported to occur more often in females, with an annual peak of 3.5/100,000 women aged 15–44 years [7].

Obesity is widely recognized as a significant risk factor for primary IIH, and recent work uncovered that risk is further associated with rapid weight gain even when these occur below the obesity threshold [5]. Secondary IIH (also known as pseudotumor cerebri) is a clinical syndrome that is similar to IIH but induced by a single identifiable cause, such as the use of steroids [4, 16], hormone replacement therapy, and oral contraception [8]. It has also been sporadically found that intracranial pressure may be increased by vancomycin [5, 9] and other antibiotics, although this has never been confirmed in large populations [1, 6, 19, 29]. Finally, anemia [25] and obstructive sleep apnea [15] are frequently described as possible causes of IIH (or, in such cases, secondary pseudotumor cerebri syndrome), though the possible mechanism for these remains unclear; however, one of the largest problems in the epidemiological research of this disease is that many of the factors that have been implicated with its development are, in fact, interlinked, and it is therefore difficult to isolate whether the associated factor is indeed directly related to IIH, or if it merely acts as a “collider.” This is exacerbated by the rarity of the disease which does not allow for large enough patient cohorts in order to be able to carry out sensitive analyses. As over 90% of patients with IIH are known to have obesity, it is possible that the range of morbidities that have been associated with IIH, such as obstructive sleep apnea, are variables descending from an ancestor cause of BMI, rather than independent associations.

This study sets out to describe the independent influence of demographic, clinical, and treatment factors on IIH prevalence in the UK population with obesity by means of a large, nationwide primary care clinical database. By introducing a population restriction to only those with a diagnosis of obesity, this study aims to address whether the development and clinical presentation of IIH in patients with obesity is independently related to a range of comorbidities that have previously been associated with IIH. Furthermore, we set out to explore the effect of BMI on the risk of IIH beyond the threshold of obesity, to explore whether the risk continues to increase with BMI in this population as has been observed in populations with a BMI up to 30 kg/m^2^.

## Methods

### Study population

The analyzed data was extracted from the Clinical Practice Research Datalink (CPRD) database, a non-profit governmental research service funded by the National Institute for Health Research (NIHR) and the Department of Health (DoH) whose design, implementation, and maintenance have been previously described [24]. Anonymized primary care public health research records, including the medical codes of hundreds of thousands of UK patients, have been entered in this database since 1987. The 123,760,872 records used for the purposes of this study were restricted to those relating to 414,522 patients with a coded clinical diagnosis of obesity (a body mass index [BMI] of ≥ 30 kg/m^2^). After excluding repeat entries and erroneous age, BMI, and weight restriction values, the analysis was based on the records of 231,399 patients.

### Ethical approval

Scientific approval for the study was granted from the Regulatory Agency’s Independent Scientific Advisory Committee and ethical approval by the Health Research Authority IRAS Project ID: 203143. ISAC approval registration number 16_140R2. For this type of study formal consent is not required.

### Data analysis

The demographic, clinical, and therapeutic factors considered were age and gender; BMI category; hypertension; hyperlipidemia; type 2 diabetes (T2DM); chronic kidney disease (CKD); chronic pulmonary disease; a history of myocardial infarction (MI); obstructive sleep apnea (OSA); gastro-esophageal reflux disease (GORD); cerebrovascular accident (CVA); stroke; peripheral vascular disease (PVD); smoking; anemia; polycystic ovarian syndrome (PCOS); and the use of steroids, non-steroidal anti-inflammatory drugs (NSAIDs), and hormone replacement therapy (HRT). The continuous variables are given as mean values ± standard deviation (SD) if normally distributed, or as median values with interquartile ranges; the categorical variables are given as relative frequencies.

Comparisons were made between the subjects with and without IIH, with the statistical significance of the differences in the categorical variables being tested using the chi-squared test. Binary logistic regression analysis was used to evaluate the characteristics associated with IIH, considering IIH the dependent variable; BMI the independent variable; and sex, age, smoking status, co-morbidities, and treatments as co-variates.

The data were extracted, prepared, and analyzed using the Statistical Package for Social Sciences, version 24 (SPSS Inc., Chicago, IL, USA) [13].

## Results

### Study population

Six hundred and seven patients had a clinical diagnosis of IIH in the cohort, translating to an overall cohort prevalence of 0.26% or 262/100,000. The average time interval between the first recorded diagnosis of obesity (BMI > 30 kg/m^2^) and the first diagnosis of IIH (available for 383 patients) was calculated for all those with a new diagnosis of obesity within the dataset (i.e., excluding those who entered the dataset with already a prior diagnosis of obesity). The average time interval was 103.6 + 70.8 months. Most of the patients (*n* = 95,902) were in the BMI category 30–35 kg/m^2^; the BMI category with the smallest number of patients (*n* = 710) was > 60 kg/m^2^. Table 1 shows the differences in the demographic, clinical and treatment characteristics of the patients with or without IIH.

**Table 1:** Demographic, clinical, and treatment characteristics in patients with or without idiopathic intracranial hypertension (*= *p* ≤ 0.05)

Characteristics		IIH (*n* = 607)	No IIH (*n* = 230,792)	*p*
Demographic				
Age*		46.5 + 15.7 years	44.6 + 15.6 years	*p* = 0.018
Gender	Male (*n* = 76,121)	205 (33.8%)	75,916 (32.9%)	
	Female (*n* = 155,268)	401 (66.2%)	154,867 (67.1%)	*p* = 0.59
Clinical				
BMI category*	30–35 (n = 95,902)	243 (48%)	95,659 (41%)	
	35–40 (*n* = 82,591)	188 (37%)	82,403 (36%)	
	40–45 (*n* = 33,356)	52 (10.1%)	33,304 (14%)	
	45–50 (*n* = 12,629)	19 (3.7%)	12,610 (5.5%)	
	50–55 (*n* = 4434)	5 (0.98%)	4429 (1.91%)	
	55–60 (*n* = 1648)	2 (0.39%)	1646 (0.71%)	
	> 60 (*n* = 710)	1 (0.19%)	709 (0.31%)	*p* < 0.0001
Hyperlipidemia		14 (2.3%)	5181 (2.2%)	*p* = 0.919
Hypertension		103 (17%)	36,704 (156%)	*p* = 0.474
Diabetes		40 (6.6%)	16,192 (7.0%)	*p* = 0.681
Smoking		226 (37%)	81,267 (35%)	*p* = 0.298
Obstructive sleep apnea		4 (0.7%)	2204 (1.0%)	*p* = 0.454
Gastro-esophageal reflux disease GORD		22 (3.6%)	8109 (3.5%)	*p* = 0.882
Peripheral vascular disease*		8 (1.3%)	1124 (0.5%)	*p* = 0.011
Polycystic ovarian syndrome*		2 (0.3%)	3066 (1.3%)	*p* = 0.013
History of myocardial infarction*		11 (1.8%)	1970 (0.9%)	*p* = 0.017
Chronic pulmonary disease		72 (11.9%)	24,529 (10.6%)	*p* = 0.179
Chronic kidney disease		10 (1.6%)	3995 (1.7%)	*p* = 0.519
History of stroke		3 (0.5%)	1970 (0.9%)	*p* = 0.240
Anemia*		120 (19.8%)	31,657 (13.7%)	*p* < 0.001
Treatment				
Non-steroidal anti-inflammatory use*		453 (74.3%)	158,277 (68.6%)	*p* = 0.001
Hormone replacement therapy use		69 (11.4%)	21,945 (9.5%)	*p* = 0.068
Steroid use*		176 (29.0%)	56,216 (24.4%)	*p* = 0.005

* Used to indicate statistical significance

### Demographic factors

The mean age of all patients with IIH in the dataset was 57.4 + 14.1 years, and their mean weight 105.5 + 17.4 kg. Of the 606 whose gender was recorded, 401 (66.2%) were female and 205 (33.8%) male.

The prevalence of IIH steadily increased with age; from 0.22% in the 18–30 age group to 0.33% in the > 70 years age group. This increase was statistically significant (*p* = 0.018). The population prevalence of IIH was similar among the females and males: 401 of 155,268 females (0.26%) and 205 of 76,121 males (0.27%) had a diagnosis of IIH; the difference was not significant (*p* = 0.59).

### Clinical factors

**Fig. 1:**
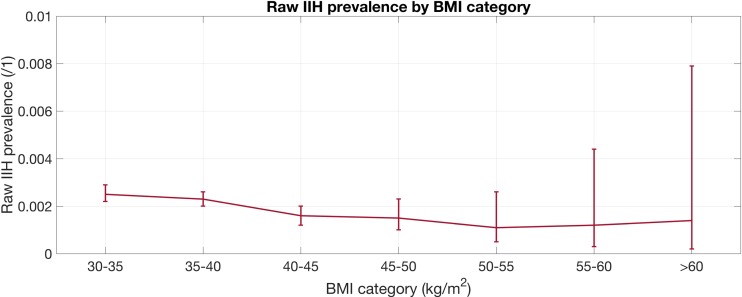
Raw prevalence of IIH by BMI category with 95% CI (p = 0.001)

**Fig. 2:**
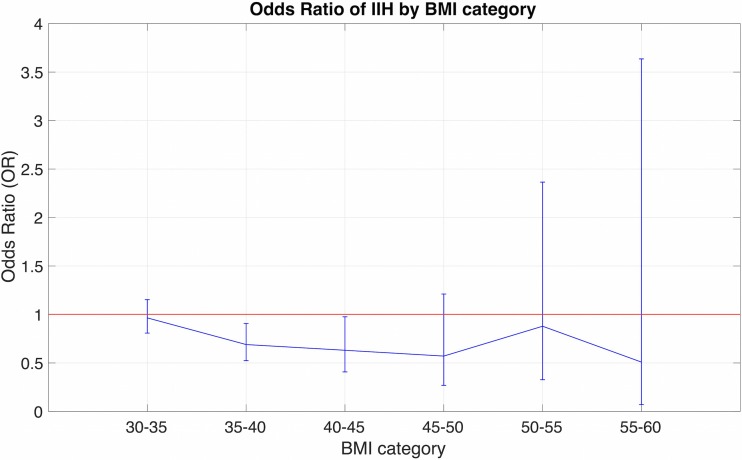
Odds ratio of developing IIH by BMI class with 95% CI (*p* = 0.050)

The mean BMI of the IIH patients was 38.2 + 5.10 kg/m^2^, and there was a steady and statistically significant (*p* = 0.001) decrease in the prevalence of IIH as BMI increased (Fig. 1). The patients with IIH had significantly higher rates of peripheral vascular disease (1.3 vs 0.5%, *p* = 0.011), ischemic heart disease (4.9 vs 3.4%, *p* = 0.021), myocardial infarction (1.8 vs 0.9%, *p* = 0.017), and anemia (19.8 vs 13.7%, *p* < 0.001) than those without, but a lower prevalence of polycystic ovarian syndrome (0.2 vs 1.3%, *p* = 0.013) (Fig. 2).

### Treatment factors

The IIH patients were more likely to have received repeat prescriptions for NSAIDs (74.3 vs 68.6%, *p* = 0.001) and steroids (29.0 vs 24.4%, *p* = 0.005).

### Multivariate analysis

**Fig. 3:**
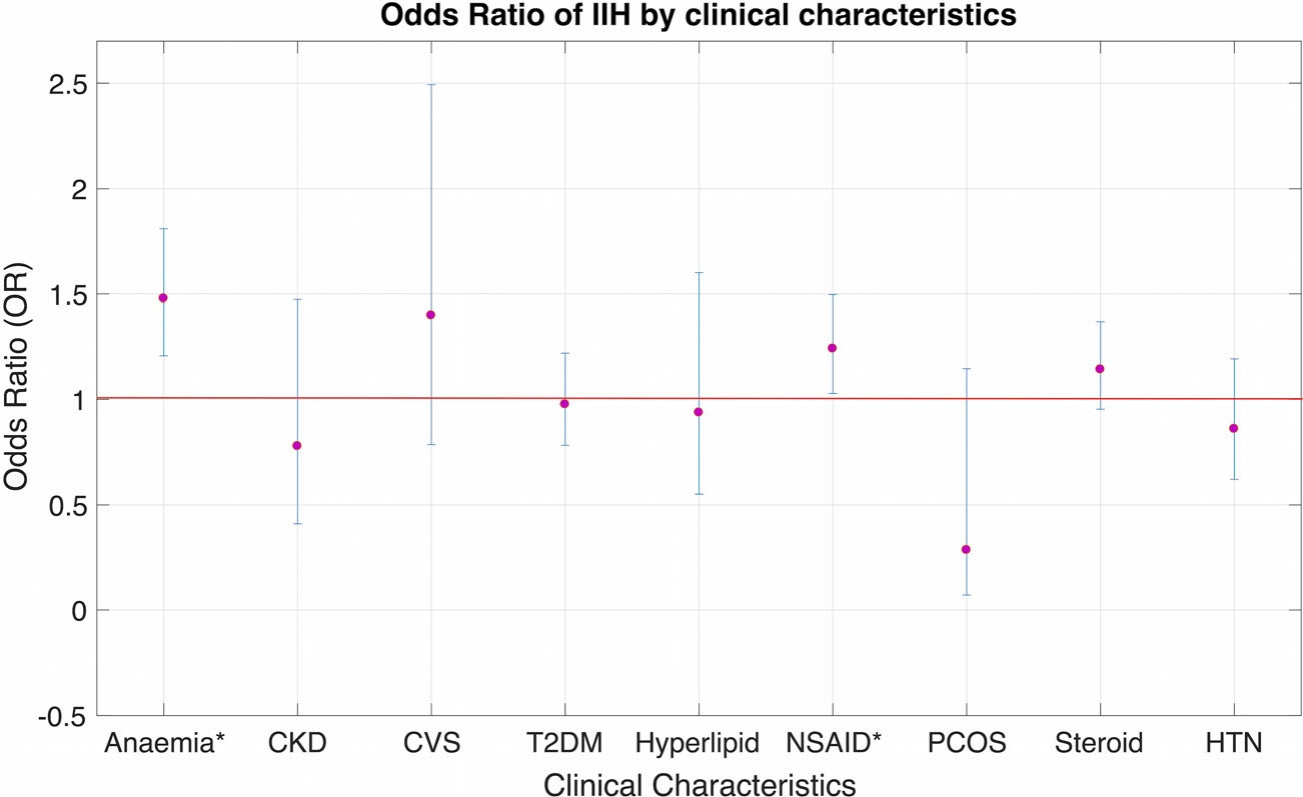
Odds ratio of developing IIH by clinical characteristics with 95% CI

Table 2 shows the results of the multivariate analysis. A history of anemia (OR = 1.478 *p* < 0.0001) and NSAID use (OR 1.241, *p* = 0.024) was independently associated with the development of IIH (Fig. 3). BMI was not associated with an increased risk of IIH on multivariate analysis after adjustment for other covariates (Fig. 2). As can be noticed in Fig. 3 and Table 2, across BMI classes, a small reduction in risk is actually observed in BMI classes of 45–50, after which the error becomes too large to accurately estimate the effect; however, this effect is non-significant overall, indicating that in this population BMI did not independently correlate with risk of IIH.

**Table 2:** Multivariate analysis of demographic, clinical, and treatment characteristics associated with IIH (*p* ≤ 0.05)

Variables	RR	95% CI		*p*
		Lower	Upper	
Age category				
Age 18–30	0.921	0.703	1.206	0.024
Age 31–40	1.203	0.936	1.546	0.172
Age 41–50	1.124	0.859	1.665	0.552
Age 51–60	1.236	0.918	1.897	0.093
Age > 60	1.303	0.895	1.601	0.106
				0.223
BMI category*				
30–35 (*n* = 95,902)	0.965	0.808	1.153	0.697
35–40 (*n* = 82,591)	0.690	0.524	0.907	0.008
40–45 (*n* = 33,356)	0.631	0.408	0.976	0.039
45–50 (*n* = 12,629)	0.571	0.269	1.211	0.144
50–55 (*n* = 4434)	0.879	0.327	2.365	0.799
55–60 (*n* = 1648)	0.509	0.071	3.636	0.501
> 60 (*n* = 710)^a^	–	–	–	–
				0.050
Hyperlipidemia	0.937	0.549	1.601	0.813
Hypertension	0.976	0.781	1.220	0.831
Type 2 diabetes	0.859	0.619	1.193	0.364
Cardiovascular disease	1.399	0.785	2.493	0.254
Chronic kidney disease	0.777	0.410	1.474	0.440
PCOS	0.285	0.071	1.146	0.077
NSAID use*	1.241	1.028	1.497	0.024
Steroid use	1.142	0.953	1.369	0.149
Anemia*	1.478	1.207	1.810	< 0.0001

* Used to indicate statistical significance

^a^Multivariate analysis could not be carried out due to low patient numbers (*n* = 1)

## Discussion

Various studies have found that obesity, defined as a BMI > 30 kg/m^2^, is associated with IIH [5, 23]. Indeed, the prevalence of IIH in this study population, restricted to patients with a diagnosis of obesity only, was estimated as being 262/100,000, which is significantly higher than the previously reported general population prevalence of 10.9/100,000 [21]. There may be a number of reasons for this. Firstly, it may, in part, be due to the inclusion criteria applied to the baseline population. This study examines databases specifically pertaining to patients with a background of obesity, in whom the prevalence of IIH may be intrinsically higher than that of the general population; however, previous studies have previously explored similar “concentrated” subsets, with Raoof et al. [21] reporting the highest subgroup prevalence of 85.7/100,000 in women with obesity. This is still lower than that found in the present study. A further explanation for this may be related to stringency of diagnostic criteria. The CPRD records are GP-based records where the diagnostic criteria applied may be variable and at times loose; though the modified Dandy criteria are most often used as the gold standard for diagnosis and most patients are likely to have been diagnosed in the secondary care setting and followed-up in primary care. Finally, another important explanation for this is that with the nature of recording in the CPRD database, most patients will not have an “end” to their diagnosis. Because of this, all patients diagnosed during the 25 years of follow-up will contribute to the “prevalence” at the end of the database entries. This may therefore overestimate the prevalence as some of the patients entered may not indeed still have the disease at this time.

Very few studies have used large-scale databases to explore the factors associated with IIH, but the association between IIH and BMI is perhaps the most widely investigated [5, 23]. It has previously been reported that as many as 90% of patients with IIH are obese [26], and many studies have found associations between IIH and female gender [7] and morbidities such as PCOS, which are inherently associated with obesity and weight gain themselves, and therefore likely to be linked to BMI rather than play a direct causal role [10]. Our study, carried out on exclusively obese patients, did not find any such relationship with female gender and PCOS; this may have been due to the fact that as many as 67.3% of the obese patients in the CPRD are women. It is therefore possible that the findings of previous studies are at least partially due to the higher rate of obesity in females. The selection criteria for this study was designed to specifically address the presentation of the disease in patients with obesity; this may have effectively annulled the “obesity bias” that can be present when looking for associations with IIH in the general population without adjusting for BMI, especially when considering patient cohorts too small to carry out sensitivity analyses or multivariate models; however, the lack of this association may also arise from diagnostic inaccuracy and the lack of central specified diagnostic criteria for IIH within the dataset. As mentioned, this is a potential limitation of the study that arises from its retrospective nature.

The association between IIH and BMI was thoroughly explored in a large case-control study by Daniels et al. [5], which found a higher incidence of IIH in patients with a higher BMI when the patients were stratified on the basis of BMI categories of < 25, 25–29, 30–35 and > 35 kg/m^2^. The results of this study showed a linear trend, which has led to the general conclusion that the incidence of IIH is likely to increase linearly with BMI.

However, our findings indicate that this linearity does not continue when considering BMI classes above 30–35 kg/m^2^; there may therefore not be a linear or “dose-response” relationship between IIH and BMI above a certain threshold. This tapering off may be partially related to the finding of Daniel et al. that rapid weight gain is more closely associated with IIH than weight itself, which is why IIH can oftentimes be diagnosed in patients without obesity, but after a moderate-to-high weight gain. Weight fluctuations are more frequent in patients with a BMI on the lower end of those considered in this study, in whom physical activity and motivation are more likely to be preserved. Furthermore, a significant proportion of the patients reporting weight gain in the study by Daniel et al. stated that it was related to pregnancy, and BMI is unlikely to increase to extreme levels such as > 60 kg/m^2^ over a period of 9 months.

This study found an independent association between IIH and anemia in patients with obesity. This is in line with finding of one consecutive case note review that eight out of 77 patients with a clinical diagnosis of IIH had documented microcytic anemia [2]. The symptoms of seven of these patients were resolved by simply correcting the anemia, thus highlighting the importance of obtaining full blood counts in patients with IIH and starting appropriate treatment if they prove to be anemic. Furthermore, the symptoms of two IIH patients in an interventional case series who did not respond to other treatment improved after blood transfusions and iron replacement therapy [17].

As in previous studies [12], our univariate analysis revealed an association between steroids and IIH but, as this disappeared in the multivariate analysis, it may be an insignificant association. It has previously been reported that there is an association between IIH and steroid withdrawal [14, 18], but we did not investigate whether the disease was diagnosed during or after steroid treatment. We did not find any association between hormone replacement therapy and IIH, but there was an independent relationship between IIH and the use of NSAIDs. This may not be causal because patients with IIH suffer from frequent and debilitating headaches for which the mainstay of treatment is analgesia, but a possible alternative mechanism underlying the relationship may involve the reduced glomerular filtration and fluid retention related to the use of NSAIDs. Further work is certainly warranted to explore this relationship in more detail. Furthermore, the specific role of other medications that were not investigated in this study—such as Vitamin A derivatives, oral contraceptive pills, tetracyclines, and other analgesics—is an important field for further research.

This study has some limitations. The CPRD is a purely retrospective database and may include errors related to losses to follow-up, incorrect entries, variations in terminology, and observer bias. Furthermore, the data and patient histories depend on precise consultation notes and documentation, which may not be complete, and the analysis includes data from patients in a single country and so the findings may not be globally generalizable. The clinical diagnoses entered in the CPRD are also dependent on consultation. Patient records may either be updated by practice employees following letters from the hospitals (which are generally copied to general practitioners) or by general practitioners themselves upon follow-up visits in primary care. It is, however. Clearly, this implies that there is room for loss of follow-up, with some patients receiving a diagnosis which is not documented in primary care records if never followed-up in that setting. Furthermore, there is a time delay between initial diagnosis in secondary or tertiary care; and the updating of patient records themselves. The date of diagnosis in the CPRD may therefore not correspond to the date of true diagnosis. Furthermore, data on the BMI of patients is reliant on regular follow-up within the primary care settings, including weight measurement. The frequency of this may be variable from patient to patient, and from practice to practice.

Perhaps the most important limitation to this study rests in the patient cohort restriction, which prevents generalizability to the overall population. The patients considered all had a BMI > 30 kg/m^2^. This was done in order to explicitly explore the trends in IIH within the population with obesity; however, this restriction may give rise to selection bias, otherwise known as “collider” bias [3], a topic which has been widely investigated especially in research in the field of obesity in relation to “obesity paradox” studies. Though stratification has been shown to introduce bias in studies, it is important to note that the size of bias introduced by colliders has been shown to be small relative to the causal relationships between the variables [22], and is significantly lower than that present when not adjusting for confounding variables to avoid the risk of a “collider” [11] as may have been the case in studies that lacked adjustment for BMI when observing trends related to IIH. Relevant to this particular work, Pizzi et al. carried out a simulation to evaluate the effect of stratification by collider factors within epidemiological cohort studies restricted to one population stratum for analysis, as this study. Very limited bias was observed [20] and the results have been further validated in numerous studies since then [27, 28]. Therefore, though an important limitation to the study, the restriction to BMI > 30 kg/m^2^ is unlikely to have introduced significant bias in the results, and does not impact the validity of the findings when considered within the context of patients with obesity only. On the other hand, the stratification by BMI is likely to have controlled for bias that has previously been reported by associating covariates that are parent variables to BMI and not directly to IIH, without adjustment for the effect being mediated by BMI as a descendant, or intermediate variable; however, as a consequence of the selection strategy, it is important to interpret these results not as crude associations between the variables (i.e., anemia and IIH) in the general population, but as associations specific to the strata of BMI studied here, therefore restricted to population with BMI > 30 kg/m^2^. We must therefore consider that there is a possibility that the associated variables of BMI, anemia, and NSAID use may not have a common ancestor with IIH or direct causal relationship outside the specific population with obesity.

### Conclusion

In conclusion, IIH is clearly a multifactorial disease occurring in patients with a vast background of co-morbidities. We have explored the relationship between obesity and IIH prevalence in a British population, stratifying patients beyond the obesity threshold, and have found that the previously proved linear trend across BMI categories that peaks at the BMI 30–35 class tails off; and no increase in risk of IIH is observed in populations with BMI increases beyond it. Anemia was the only clinical factor to be independently associated to IIH; and NSAID use the only treatment factor. Due to selection criteria, however, the results of all associations or lack thereof are not generalizable to the populations without obesity; therefore, future studies are certainly needed to characterize the relationship between BMI and the development of IIH over an unrestricted population, with careful adjustment. Further investigations on the mechanism behind the elusive link between IIH and clinical characteristics such as anemia and NSAID use in populations with obesity are also warranted.

## References

[1] Bruce BB, Kedar S, Van Stavern GP, Monaghan D, Acierno MD, Braswell RA, Preechawat P, Corbett JJ, Newman NJ, Biousse V (2009). Idiopathic intracranial hypertension in men. Neurology.

[2] Chagot C, Blonski M, Machu J-L, Bracard S, Lacour J-C, Richard S (2017). Idiopathic intracranial hypertension: prognostic factors and multidisciplinary management. J Obes.

[3] Cole SR, Platt RW, Schisterman EF, Chu H, Westreich D, Richardson D, Poole C (2010). Illustrating bias due to conditioning on a collider. Int J Epidemiol.

[4] Dandy WE (1937). Intracranial pressure without brain tumour: diagnosis and treatment. Ann Surg.

[5] Daniels AB, Liu GT, Volpe NJ, Galetta SL, Moster ML, Newman NJ, Biousse V, Lee AG, Wall M, Kardon R, Acierno MD, Corbett JJ, Maguire MG, Balcer LJ (2007) Profiles of obesity, weight gain, and quality of life in idiopathic intracranial hypertension (Pseudotumor Cerebri). Am J Ophthalmol 143(4). 10.1016/j.ajo.2006.12.040 10.1016/j.ajo.2006.12.040 17386271

[6] Deonna T, Guignard JP (1974). Acute intracranial hypertension after nalidixic acid administration. Arch Dis Child.

[7] Durcan FJ, Corbett JJ, Wall M (1988). The incidence of pseudotumor cerebri. Population studies in Iowa and Louisiana. Arch Neurol.

[8] Finsterer J, Kues EW, Brunner S (2006). Pseudotumour cerebri in a young obese woman on oral contraceptives. Eur J Contracept Reprod Heal Care.

[9] Gaskill SJ, Marlin AE (1992). Vancomycin: its effect on intracranial pressure. Pediatr Neurosurg.

[10] Glueck CJ, Aregawi DA, Goldenberg N, Golnik KC, Sieve L, Wang P (2005). Idiopathic intracranial hypertension, polycystic-ovary syndrome, and thrombophilia. J Lab Clin Med.

[11] Greenland S (2003). Quantifying biases in causal models: classical confounding vs collider-stratification bias. Epidemiology.

[12] Greer M (1968). Management of benign intracranial hypertension (pseudotumor cerebri). Clin Neurosurg.

[13] IBM Corp (2016). IBM SPSS statistics for Windows, Version 24.0.

[14] Kaul B, Ramanarayanan S, Mahapatra H, Sethi TK, Ahlawat R (2009) Iron deficiency masquerading as idiopathic intracranial hypertension. BMJ Case Rep. 10.1136/bcr.06.2008.0346 10.1136/bcr.06.2008.0346 PMC3028304 21686848

[15] Lavie P, Herer P, Hoffstein V (2000). Obstructive sleep apnoea syndrome as a risk factor for hypertension: population study. BMJ.

[16] Liu GT, Kay MD, Bienfang DC, Schatz NJ (1994). Pseudotumor cerebri associated with corticosteroid withdrawal in inflammatory bowel disease. Am J Ophthalmol.

[17] Mollan SP, Ball AK, Sinclair AJ, Madill SA, Clarke CE, Jacks AS, Burdon MA, Matthews TD (2009). Idiopathic intracranial hypertension associated with iron deficiency anaemia: a lesson for management. Eur Neurol.

[18] Mollan SP, Ali F, Hassan-Smith G, Botfield H, Friedman D, Sinclair A (2016). Evolving evidence in adult idiopathic intracranial hypertension: pathophysiology and management. J Neurol Neurosurg Psychiatry.

[19] Mushet GR (1977). Pseudotumor and nitrofurantoin therapy [letter]. Arch Neurol.

[20] Pizzi C, De Stavola B, Merletti F, Bellocco R, dos Santos Silva I, Pearce N, Richiardi L (2011). Sample selection and validity of exposure-disease association estimates in cohort studies. J Epidemiol Community Health.

[21] Raoof N, Sharrack B, Pepper IM, Hickman SJ (2011). The incidence and prevalence of idiopathic intracranial hypertension in Sheffield, UK. Eur J Neurol.

[22] Sperrin M, Candlish J, Badrick E, Renehan A, Buchan I (2016). Collider bias is only a partial explanation for the obesity paradox. Epidemiology.

[23] Szewka AJ, Bruce BB, Newman NJ, Biousse V (2013). Idiopathic intracranial hypertension: relation between obesity and visual outcomes. J Neuroophthalmol.

[24] The Clinical Practice Research Datalink (CPRD), www.cprd.com Last accessed: 27 April 2018

[25] Tugal O, Jacobson R, Berezin S, Foreman S, Berezin S, Brudnicki A, Godine L, Davidian MM, Jayabose S, Escobedo V (1994). Recurrent benign intracranial hypertension due to iron deficiency anaemia. Case report and review of the literature. Am J Pediatr Hematol Oncol.

[26] Wall M (2010). Idiopathic intrcranial hypertension. Neurol Clin.

[27] Wei L, Brookhart MA, Schneeweiss S, Mi X, Setoguchi S (2012). Implications of m bias in epidemiologic studies: a simulation study. Am J Epidemiol.

[28] Whitcomb BW, McArdle PF (2016). Collider-stratification bias due to censoring in prospective cohort studies. Epidemiology.

[29] Winrow AP, Supramaniam G (1990). Benign intracranial hypertension after ciprofloxacin administration. Arch Dis Child.

